# Supplementary material for: SIV-induced terminally differentiated adaptive NK cells in lymph nodes associated with enhanced MHC-E restricted activity
Source: Nat Commun. 2021 Feb 24;12:1282. doi: 10.1038/s41467-021-21402-1 (PMC7904927; doi:10.1038/s41467-021-21402-1)
Supplement: Supplementary file 1 — Supplementary Information [file 41467_2021_21402_MOESM1_ESM.pdf]

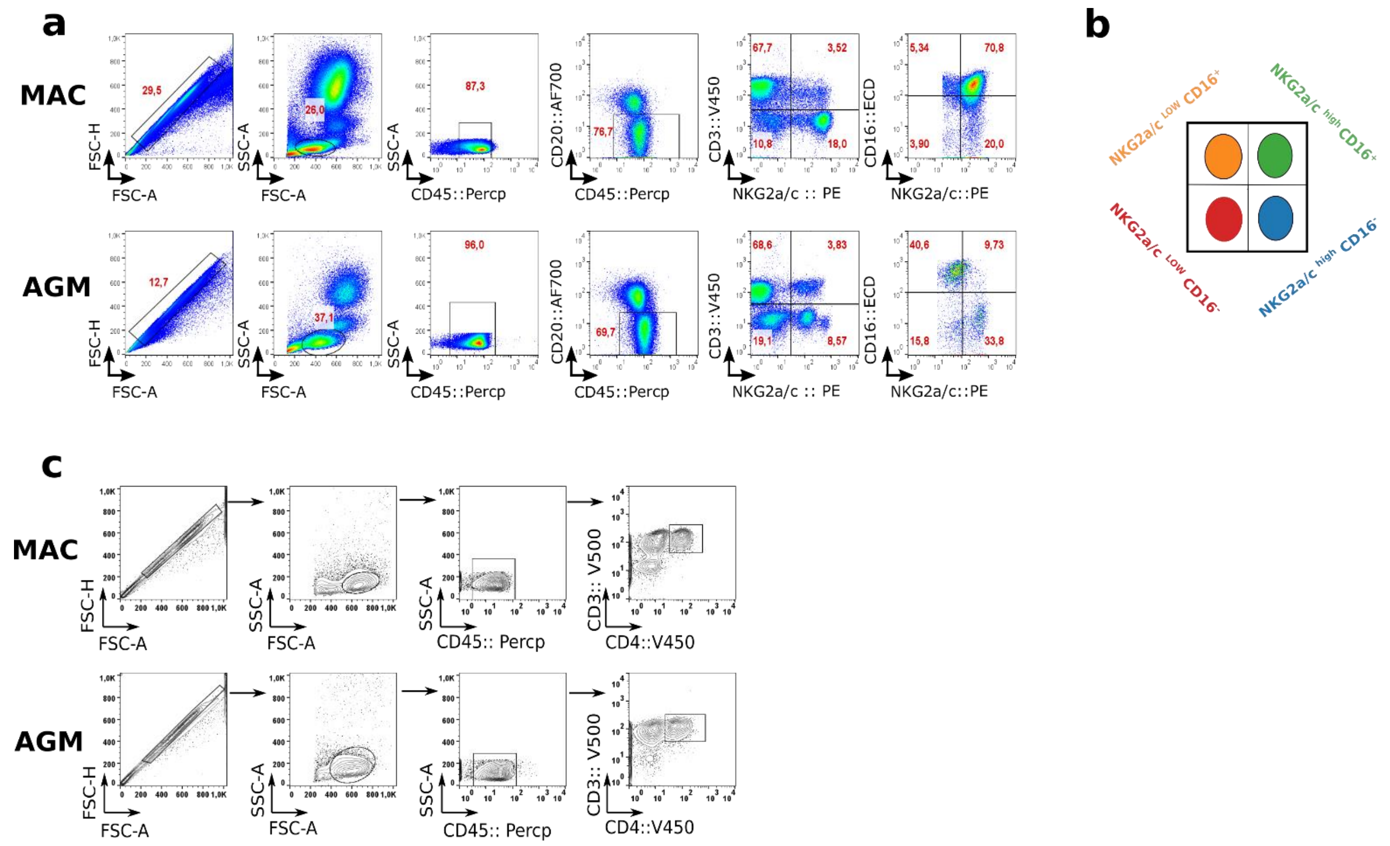

Supplementary Figure 1. Gating strategy used in the study. (**a**) Gating strategy used to define NK cell subpopulations in MAC (upper panel) and AGM (lower panel). (**b**) Scheme showing NK cell subpopulations according to $NKG2_{a/c}$ and CD16 expression. The color code used to define each of the four subpopulations is the same throughout the manuscript. (**c**) Gating strategy used to define CD4 T cells in MAC (upper panel) and AGM (lower panel).

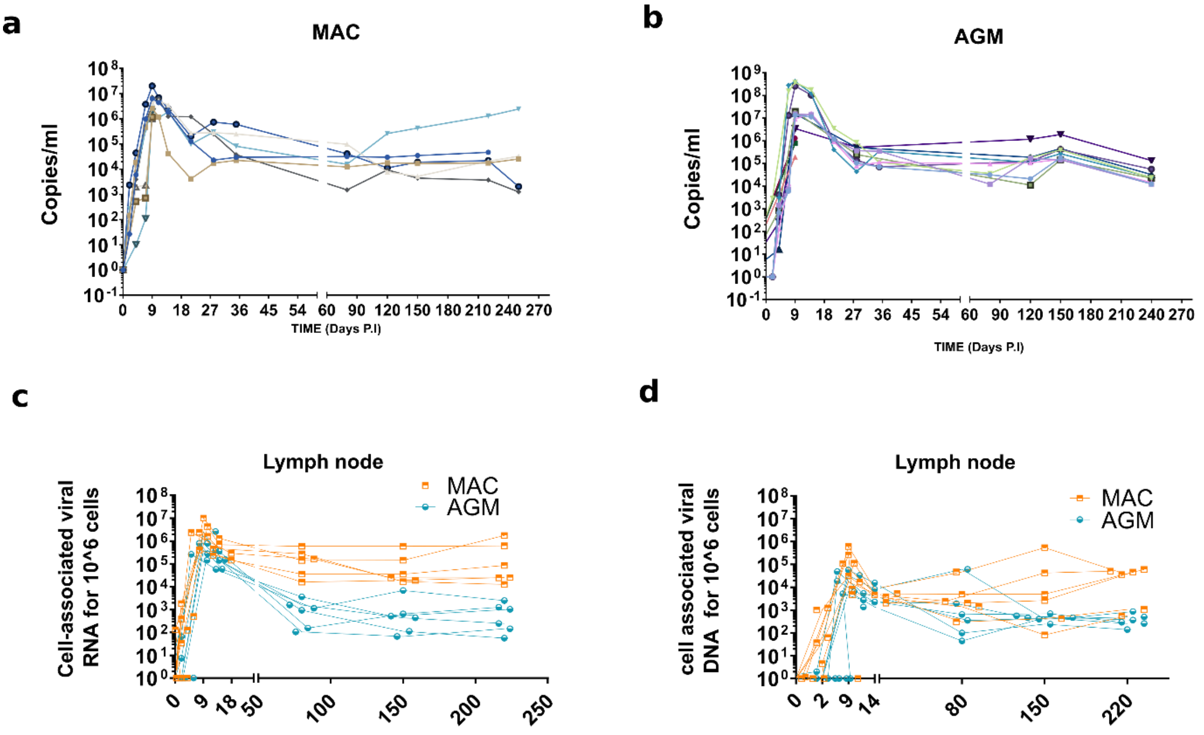

e

Animal #	Species	Duration of infection at necropsy (days)	SIV	Viral status	vRNA (copies/ml plasma)	SIV strain
RM 14222	Rh.Mac	n.a.	neg	n.a.	n.a.	n.a.
RM 2363	Rh.Mac	n.a.	neg	n.a.	n.a.	n.a.
RM 2564	Rh.Mac	n.a.	neg	n.a.	n.a.	n.a.
RM 14225	Rh.Mac	n.a.	neg	n.a.	n.a.	n.a.
AV556	Rh.Mac	n.a.	neg	n.a.	n.a.	n.a.
RM 13926	Rh.Mac	2512	+	Controller	$4.2x10^1$	SIVmac239
RM 12671	Rh.Mac	4367	+	Controller	<40	
RM 2139	Rh.Mac	4268	+	Controller	<40	
RM 2284	Rh.Mac	2483	+	Controller	<40	
RM 13919	Rh.Mac	2465	+	Controller	<40	
RM 13923	Rh.Mac	2150	+	Controller	<40	
RM2155	Rh.Mac	4373	+	Controller	4.2×10^1	
RM 2153	Rh.Mac	4107	+	Viremic	8.4×10^4	
RM 13909	Rh.Mac	2368	+	Viremic	$8.86x 10^4$	
RM 8644	Rh.Mac	6349	+	Viremic	2.3×10^4	
RM 15925	Rh.Mac	394	+	Viremic	$4.12x10^4$	
RM 13914	Rh.Mac	448	+	Viremic	$3.56x10^5$	
RM 2347	Rh.Mac	203	+	Viremic	$3.60x10^6$	

Supplementary Figure 2. Plasma viral RNA copy numbers in the longitudinally followed animals. The longitudinal studies included **(a)** 6 cynomolgus macaques (cynMAC) infected with SIVmac251 and **(b)** 6 AGM infected with SIVagm.sab92018. **(c)** Cell associated viral RNA copy numbers in lymph nodes. **(d)** Cell-associated viral DNA copy numbers in lymph nodes. **(e)** Table showing immunological parameters at necropsy of rhesus macaques used in the study. All the values are provided in the Source Data file.

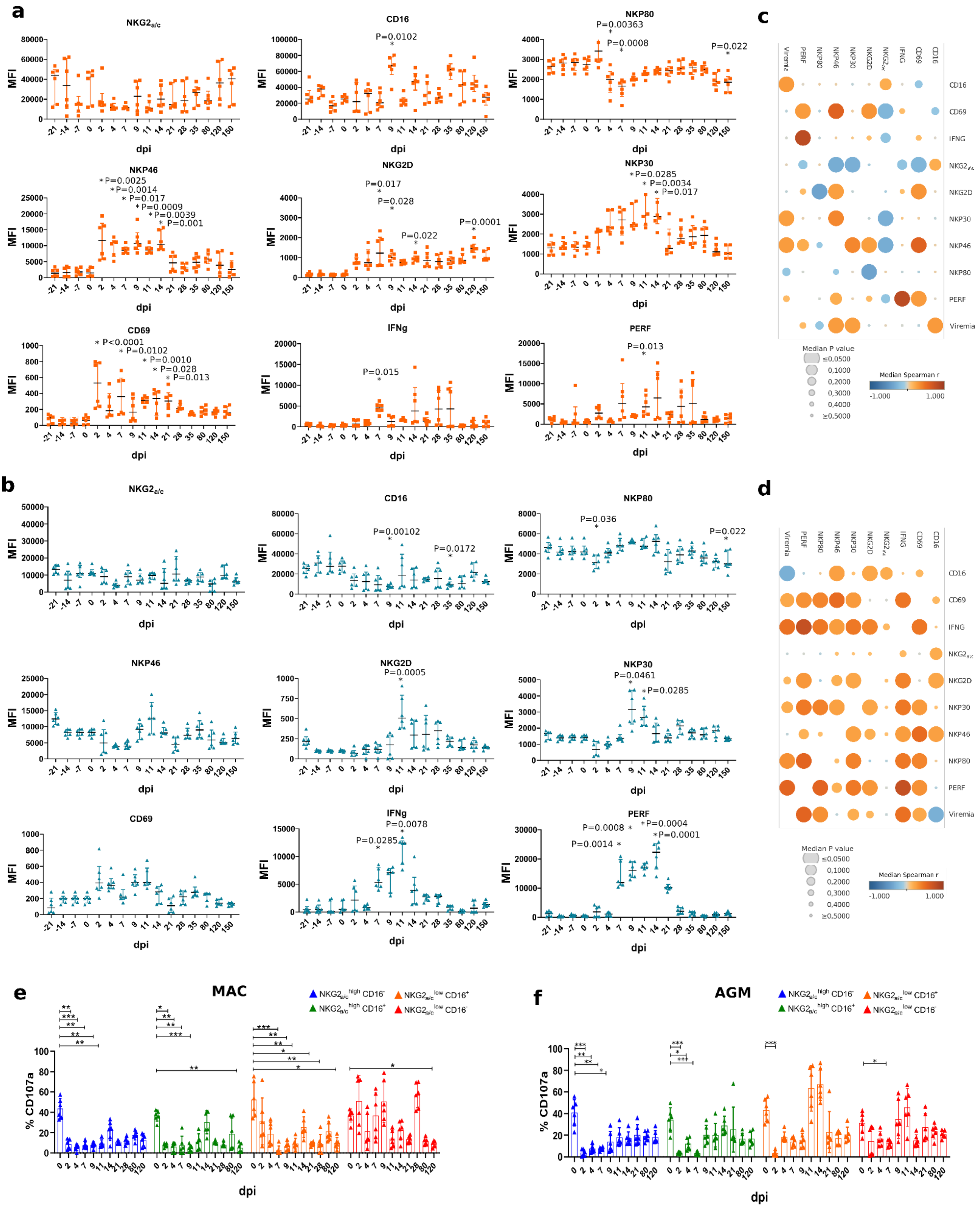

Supplementary Figure 3: Evolution of the level of blood NK cell markers during SIV infection. **(a, b)** Longitudinal analysis of the level of NK cell markers in blood during (**a**) SIVmac (orange) and (**b**) SIVagm (blue) infection. Cells were analyzed by flow cytometry from blood collected longitudinally from six animals per species. Horizontal black bars represent the median and error bars indicate interquartile range. Each symbol represents an individual animal. For group comparisons, two-sided Wilcoxon signed-rank test with Bonferroni correction were used (n=13). P values of less or equal to 0.05 were considered statistically significant. Asterix indicate a significant change when compared to the base line. Exact P values are provided on the graphs. **(c, d)** Spearman correlation matrix of NK cell expression (MFI) and measures of viremia. The Spearman correlation is shown. The matrix was built using the values obtained between day 0 and day 150 p.i from (**c**) 6 MAC and (**d**) 6 AGM. The orange and blue colors indicate, respectively, higher and lower r values. The p value is indicated by the size of the circle. **(e,f)** Functional activity of blood NK cells followed longitudinally in (**e**) MAC and (**f**) AGM. CD107a expression was measured on NK cell subsets after exposure to the MHC-I-devoid K562 target cells. Columns represent the median; the interquartile range is indicated by the error bars. Each rectangle (MAC) or triangle (AGM) represents an individual animal. Day 0 represents the median of all the time points before infection. For group comparisons two-sided Wilcoxon signed-rank test with Bonferroni correction were used (n=13). P values of less or equal to 0.05 were considered statistically significant. Asterix indicate a significant change when compared to the base line. Asterix are graphically annotated as follows: *, p<0.05; **, p<0.01; ***, p<0.001; ****, p<0.0001. All p-values and r values from panels **(c)** and **(d)** are provided in the Source Data file.

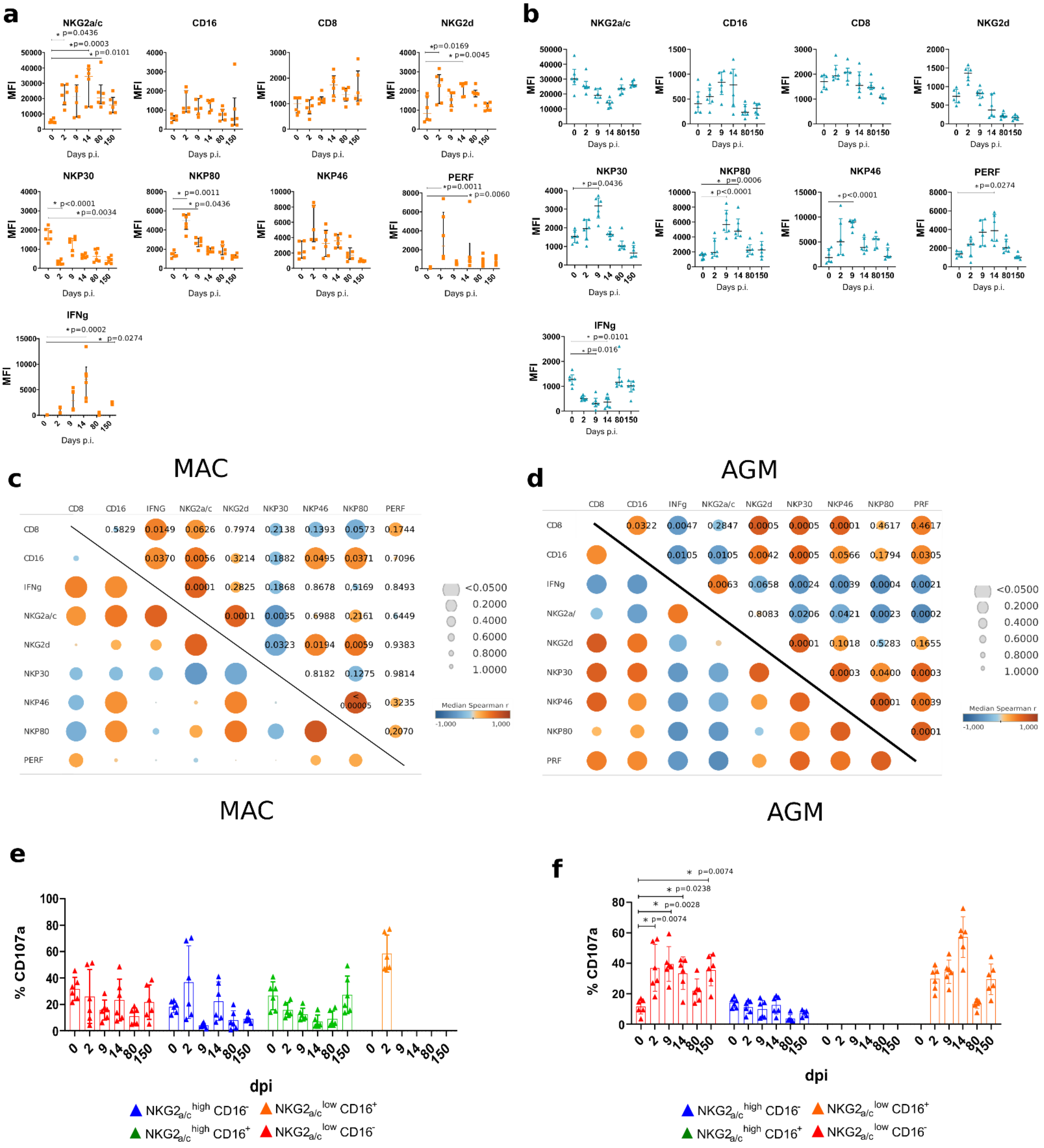

Supplementary Figure 4: Longitudinal analysis of NK cell markers in LN during SIV infections. **(a-b)** Cells were analyzed ex vivo by flow cytometry from LN collected longitudinally from **(a)** six MAC and **(b)** six AGM. Bars represent the median, error bars indicate the quantifiable range. Each symbol represents an individual animal. For group comparisons two-sided Wilcoxon signed-rank test with Bonferroni correction were used (n=6). P values of less or equal to 0.05 were considered statistically significant. Asterix indicate significant change when compared to the base line. Exact P values are provide on the graphs. **(c-d)** Spearman's correlation matrix of NK cell marker levels in LN from **(c)** MAC and **(d)** AGM. Matrixes were built using the values between day 0 and 150 p.i of **(c)** 6 MAC and **(d)** 6 AGM. The orange and blue colors indicate higher and lower r values, respectively. The p value is indicated by the size of the circle. All p-values are indicated on the figure. Rho values are listed in Suppl. Table 1. **(e-f)** Cytolytic activity of **(e)** MAC and **(f)** AGM NK cell subsets. Peripheral LN cells obtained before and during the infection were co-cultured with K562 cells. The frequency of CD107a surface expression on $NKG2_{a/c}^{low}CD16^{-}$ (red), $NKG2_{a/c}^{HIGH}CD16^{-}$ (blue), $NKG2_{a/c}^{HIGH}CD16^{+}$ (green) and $NKG2_{a/c}^{low}CD16^{+}$ (orange) NK cells is shown. Columns represent the median; error bars indicate the interquartile range. Day 0 represents the median of all the time points before infection. For group comparisons, two-sided Wilcoxon signed-rank test with Bonferroni correction were used (n=7). P values of less or equal to 0.05 were considered statistically significant. Asterix indicate significant changes when compared to the base line. Exact p values are provided on the graphs. All the values are provided in the Source Data file.

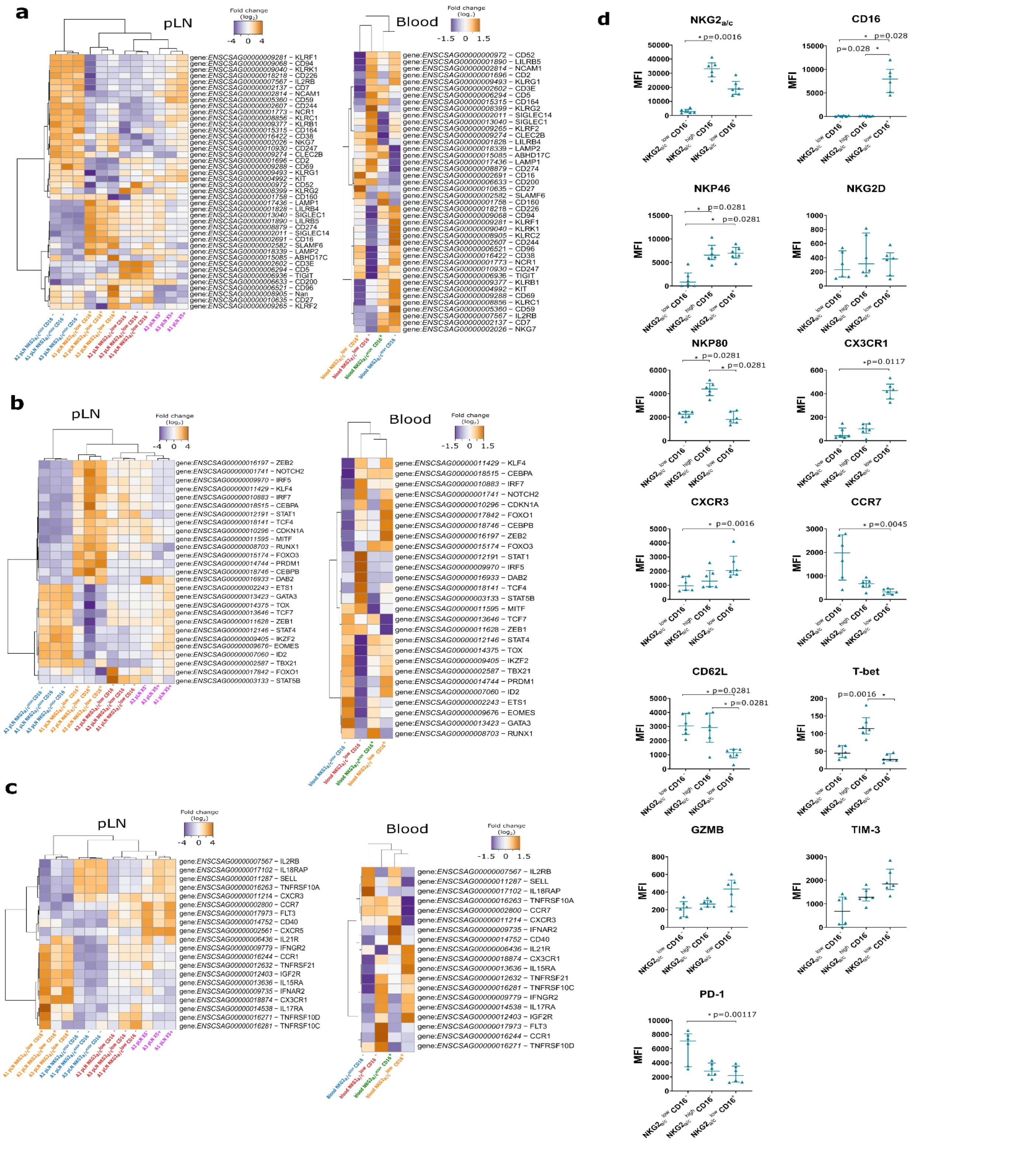

Supplementary Figure 5. Gene expression profiles in LN and blood NK cells from chronically infected AGMs. Heatmaps of the transcript signatures for **(a)** NK cell surface markers, **(b)** transcription factors**, (c)** surface receptors. In each panel, NK cell subsets are organized as in Fig.2. Each row represents a variable gene among clusters, and each column represents the median of 3 monkeys. (**d**) Mean fluorescent intensity (MFI) for a given marker on NK cells during the chronic phase (day 150 p.i.) of SIVagm infection in AGM LN. Each monkey is represented by a triangle. The black bar represents the median, the error bar shows the interquartile range. For groups comparisons two-

sidedWilcoxon signed-rank test with Bonferroni correction were used (n=3). P values of less or equal to 0.05 were considered statistically significant. Asterix indicate significant change. Exact P values are provided on the graphs.

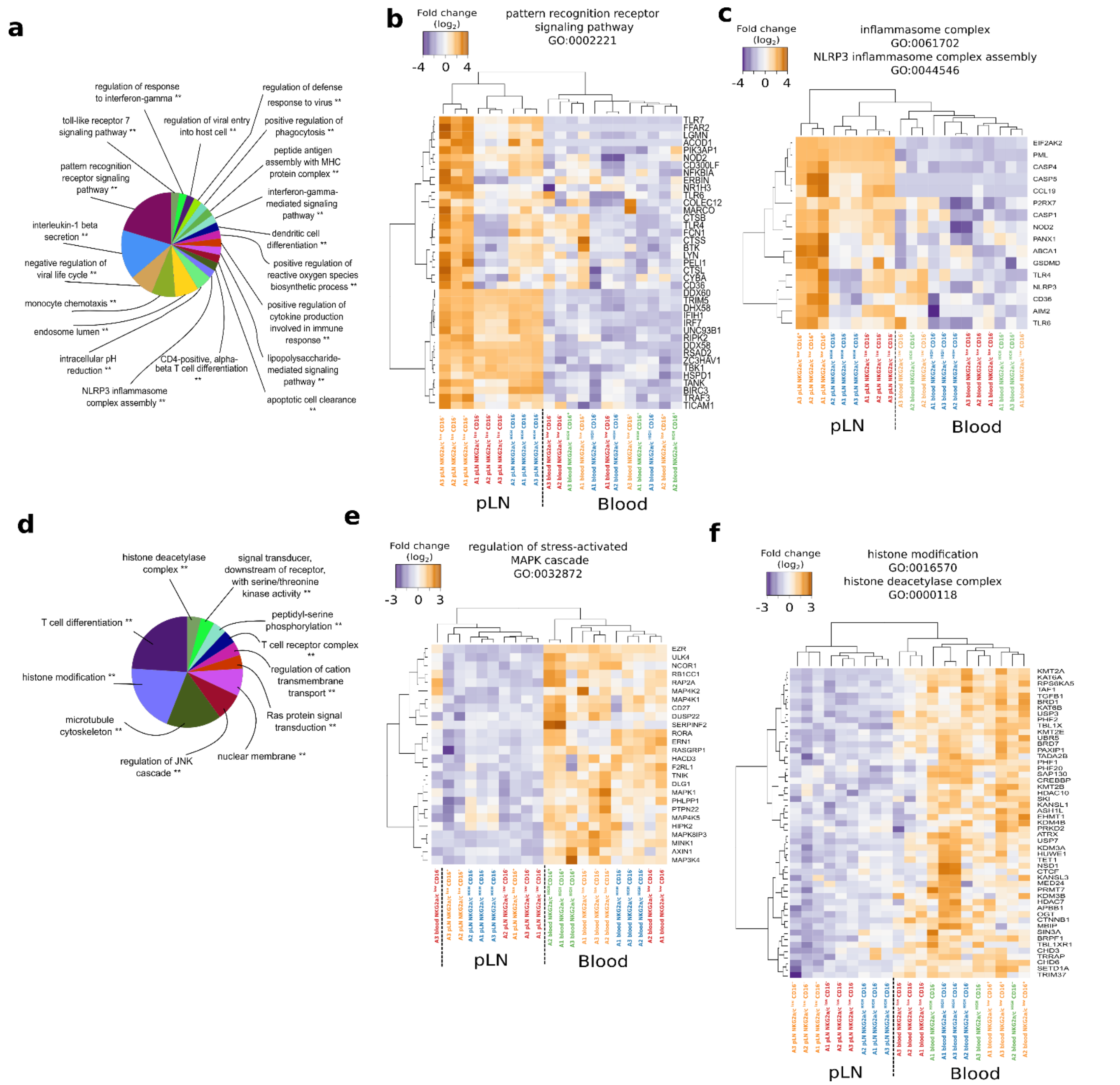

Supplementary Figure 6. Transcriptome analysis of NK cells in blood and LN from chronically SIVagm-infected AGMs. (a) The pie chart shows the enriched Gene Ontology (GO) terms for the up-regulated genes shared by all NK cell subsets in LN when compared to blood. The 'cellular component', 'biological process', 'molecular function' and 'immune system process' GO terms were selected for this analysis. The two-sided hypergeometric test was used in the statistical inference. The term value corrected with the Bonferroni step down method was applied for p-value correlation. The adjusted p-value threshold was set to 0.001. The list of the genes found for each GO term is given in the Source Data file. Each pie slice represents the percentage of genes found in a given pathway among total up-regulated genes in LN. **(b)** and **(c)** Heatmaps showing all the genes differentially expressed in blood and LN cell subsets. P values of less or equal to 0.05 were considered statistically significant. In each panel the name of the proteins corresponding to the transcripts are indicated. **(d)** The pie chart shows the enriched GO terms for the downregulated genes shared by all LN NK cells when compared to blood ones. The analysis was the same as described in **(a)**. The list of genes found in each pathway is given in the Source Data file. Each pie slice represents the percentage of genes found in a given pathway among the total genes downregulated in LN NK cells. **(e)** and **(f)** Heatmaps showing all the genes found to be significantly downregulated in LN NK cells in at least one comparison between blood and LN cell subsets. P values of less or equal to 0.05 were considered statistically significant. In each panel the name of the proteins corresponding to the transcripts are indicated. Three chronically infected monkeys were analysed. Source data have been deposited in the Gene Expression Omnibus database; the accession number is GSE140600. https://www.ncbi.nlm.nih.gov/geo/query/acc.cgi?acc=GSE140600. All the pathways obtained during the GO enrichment analysis are provided in the Source Data file.

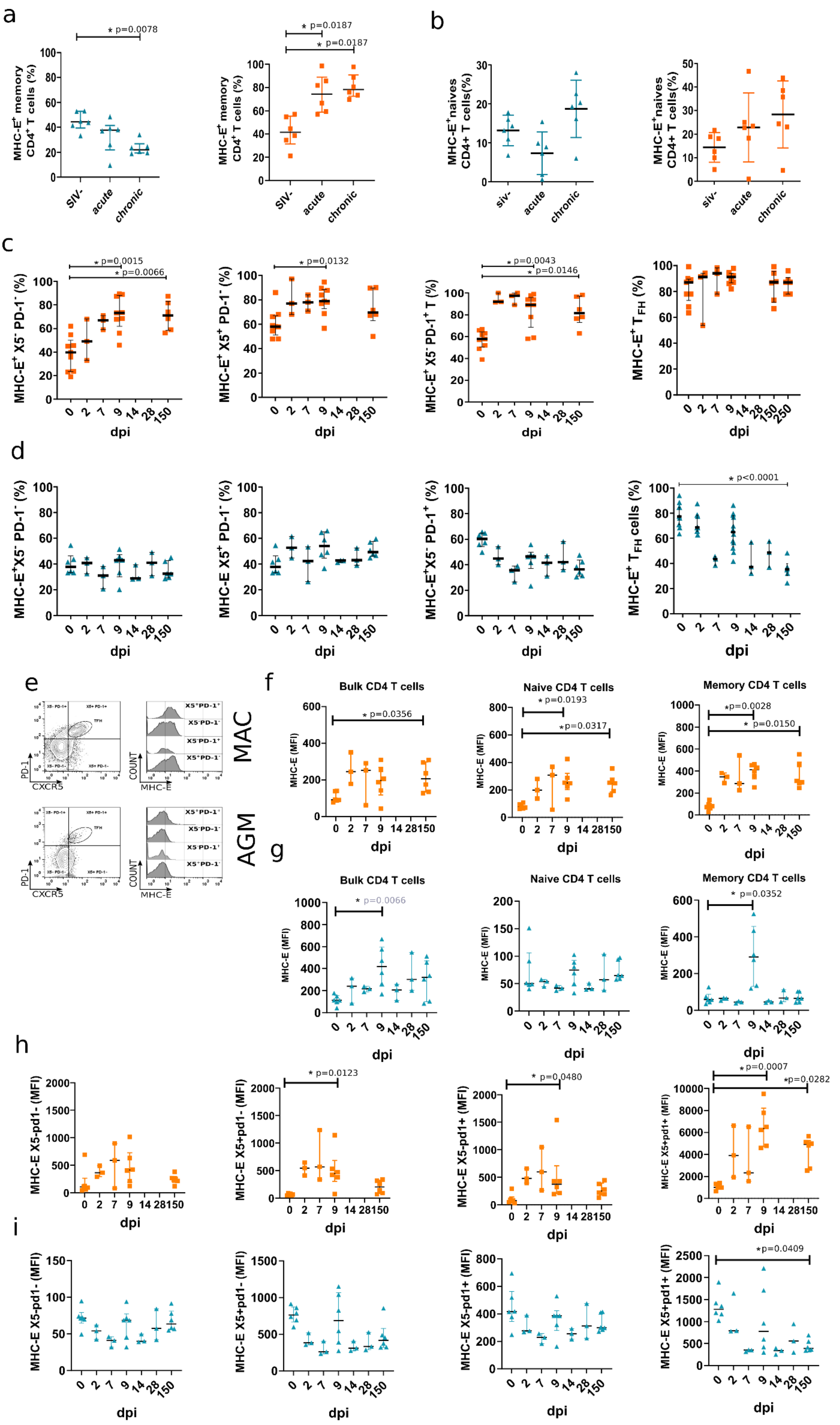
a
b
c
d
e
f
g
h
i
MHC-E+ memory CD4+ T cells (%)
MHC-E+ naives CD4+ T cells(%)
SIV-
acute
chronic
* p=0.0078
* p=0.0187
* p=0.0187
MHC-E+ X5- PD-1- (%)
MHC-E+ X5+ PD-1- (%)
MHC-E+ X5- PD-1+ T (%)
MHC-E+ TFH (%)
MHC-E+ TFH cells (%)
dpi
* p=0.0015
* p=0.0066
* p=0.0132
* p=0.0043
* p=0.0146
* p<0.0001
MAC
AGM
PD-1
CXCR5
COUNT
MHC-E
X5+PD-1+
X5-PD-1-
X5-PD-1+
X5+PD-1-
TFH
Bulk CD4 T cells
Naive CD4 T cells
Memory CD4 T cells
MHC-E (MFI)
* p=0.0356
* p=0.0193
* p=0.0317
* p=0.0028
* p=0.0150
* p=0.0066
* p=0.0352
MHC-E X5-pd1- (MFI)
MHC-E X5+pd1- (MFI)
MHC-E X5-pd1+ (MFI)
MHC-E X5+pd1+ (MFI)
* p=0.0123
* p=0.0480
* p=0.0007
* p=0.0282
* p=0.0409

Supplementary Figure 7. MHC-E expression on CD4$^+$ T cells in LN during pathogenic and nonpathogenic SIV infection. **(a-b)** Frequencies of MHC-E$^+$ cells among **(a)** memory and **(b)** naive CD4$^+$ T cells in LN from MAC (orange) and AGM (blue) before and during SIV infection. Each symbol represents an individual monkey. The black bar represents the median and error bars the interquartile range. Comparisons were done with a two-sided Wilcoxon signed-rank test with Bonferroni correction (n=3). P ≦0.05 was considered statistically significant and marked by asterixs. (**c-d)** Longitudinal analyses on MHC-E$^+$ cell frequencies among distinct memory CD4$^+$ T cell subpopulations in LN from infected MAC (orange) and AGM (blue). Each symbol represents an individual monkey. The black bar represents the median and the error bar the interquartile range. Comparisons were performed for time points where samples from 6 animals have been analyzed (days 0, 9 and 150 p.i.), using a two-sided Wilcoxon signed-rank test with Bonferroni correction (n=3). P ≦ 0.05 was considered statistically significant and indicated by asterixs. **(e)** A representative dot plot and histogram showing MHC-E expression on distinct CD4$^+$ T cell subpopulations in LN. **(f-g)** Longitudinal analyses of MHC-E expression levels on total, naive and memory CD4$^+$ T cells, in LN from infected MAC (orange) and AGM (blue). Each symbol represents an individual monkey. The analyses were performed as described for (a-b). **(h-i)** Longitudinal analyses of MHC-E expression levels on distinct CD4$^+$ T cell subpopulations in LN from infected (h) MAC (orange) and (i) AGM (blue). Each monkey is represented by a symbol. The analyses were performed as described for (a-b).

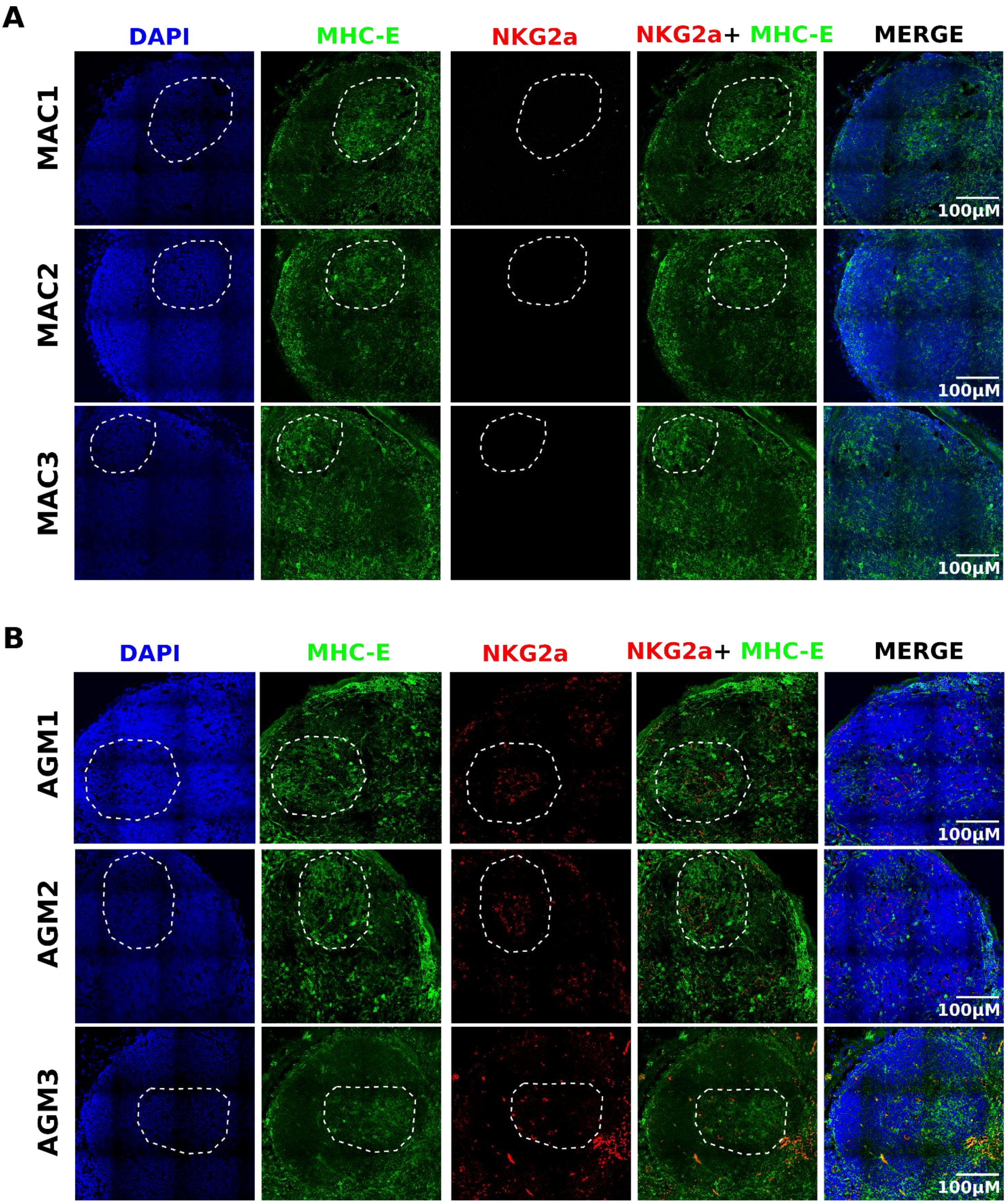

Supplementary Figure 8. **Distribution of MHC-E and NKG2a/c positive cells in B cell follicles. Peripheral LNs from six AGM and six MAC were analyzed at day 150 p.i.** The figure shows representative confocal images of LN sections from 3 MAC **(a)** and 3 AGM **(b)** stained for MHC-E (green), NKG2A (red) and total nucleus (blue).

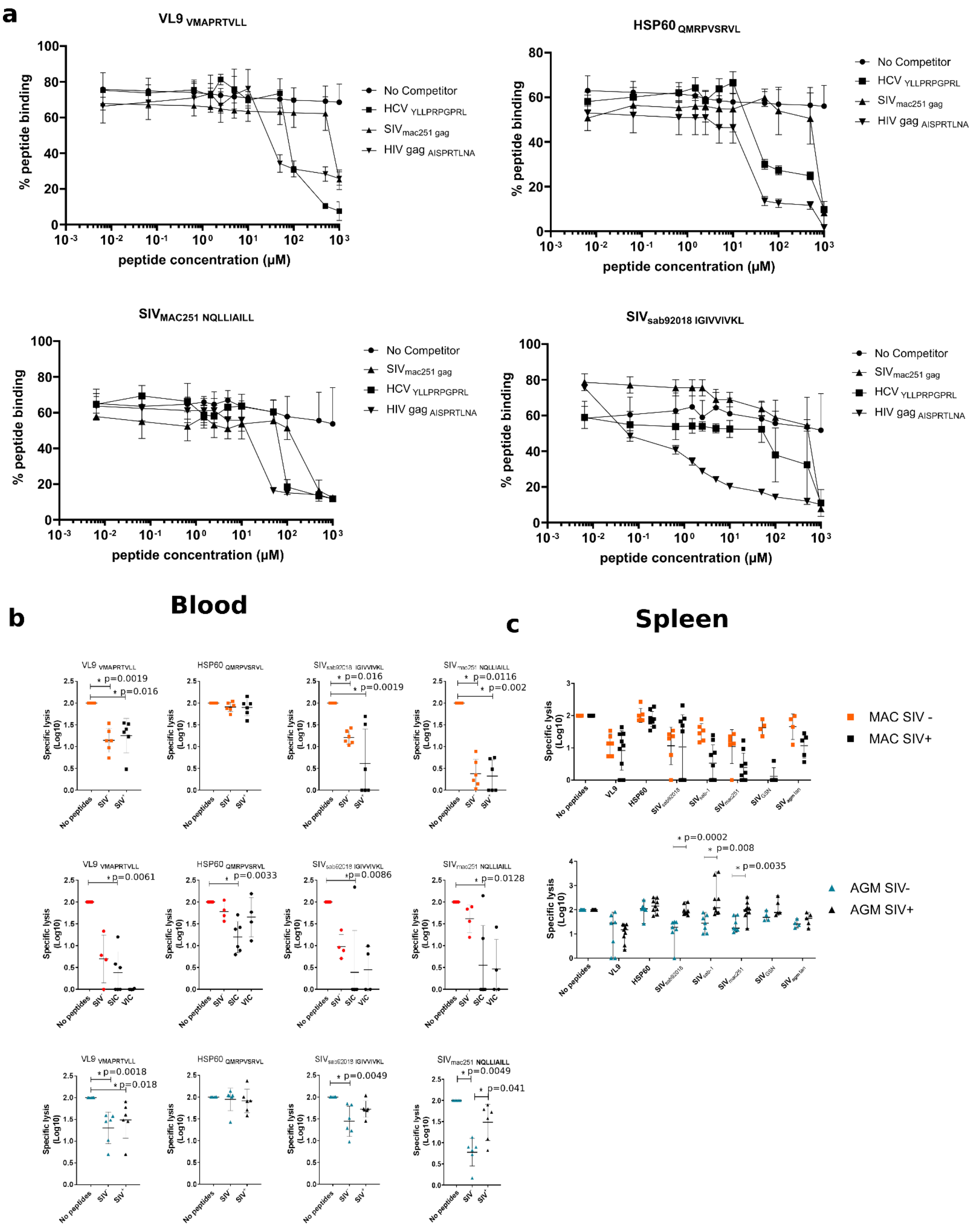

Supplementary Figure 9. MHC-E dependent cytotoxic activity of NK cells before and after SIV infection. (a) Competition assay for binding to MHC-E. K562-HLA-E*0101 cells were loaded with biotin-A2 VL9 peptides**,** biotin-HSP60 peptides**,** biotin-SIVmac ENV LS peptides and biotin-SIVagm ENV LS peptides**.** An increasing concentration of the indicated unlabeled competitor peptides was introduced into the culture. MHC-E-bound biotinylated peptide was measured at the surface of K562 cells. Data represent the median of three independent experiments. The sequence of the region coding for the SIVmac251 peptide is identical to that of SIVmac239. (**b**) MHC-E dependent cytotoxic activity of NK cells from blood before and during chronic SIV infection. Uninfected cynomolgus MAC are indicated in orange squares, uninfected rhesus MAC in red circles and uninfected AGM in blue triangles. SIV infected animals are indicated in black. Rhesus MAC corresponded to 4 uninfected, 6 controller (SIC) and 4 viremic (VIC) animals. For cynomolgus MAC and AGM, comparisons were done with a two-sided Wilcoxon signed-rank test with Bonferroni correction (n=3). P values of less or equal to 0.05 were considered statistically significant. Asterix indicate significant change when compare to the base line. Exact P value are provide on the graphs. For . Rhesus MAC, significant difference between the two conditions was determined using two-tail Mann–Whitney test . P values of less or equal to 0.05 were considered statistically significant and indicated by asterixs. **(c)** MHC-E dependent cytotoxic activity of NK cells from spleen before and during chronic SIV infection. Peptides derived from ENV LS of SIVagm.sab$_{92018}$, SIVagm.$_{sab1}$, SIVmac$_{239/251}$, SIVgsn and SIVagm.tan were analyzed. NK cell activities for uninfected cynomolgus MAC are indicated in orange squares and uninfected AGM in blue triangles. NK cell activities for SIV infected animals are depicted in black. The SIVmac251 peptide is identical to the corresponding SIVmac239 region. Each symbol represents an individual monkey. The black bar represents the median and the error bar the interquartile range. Significant difference between healthy and SIV-infected monkeys were determined using two-tail Mann–Whitney test. P values of less or equal to 0.05 were considered statistically significant and indicated by asterixs.

a

Protein	Accession	Position 1–10	11–20	21–30	31–40
HLA class I	Q31612	MLVMAPRTVL	LLLSAALALT	ETWAGSHSMR	YFHTSVSRPG
HSP60	AAA36022	MLRLPTVFRQ	MRPVSRVLAP	HLTRAYAKDV	KFGADARALM
SIV$_{MAC239}$	AAL68909	MGCLGNQLLI	AILLLSVCGI	YCTLYVTVFY	GVPAWRNATI
SIV$_{mac251}$	AGO97057	MGCLGNQLLI	AILLLSVCGI	YCTQYVTVFY	GVPAWRNATI
SIV$_{agm\ Sab-1}$	S46352	MKLLTVLLWL	SGCWSLVWLV	QYVTVFYGIP	VWKNSSVQAF
SIV$_{agm\ Sab92018}$	ADO34206	MKLLFLLVFL	IGIVVIVKLE	QYVTVFYGIP	VWQNSSVQAF
SIV$_{smSL92b}$	AAK55281	MACPGLHLLI	DILFLSVLGT	WCAQYVTIFY	GIPAWRNATI
SIV$_{agm.tan-1}$	AAC57057	MKLLILWLVI	GLGVGIVQLT	QYVTVFYGIP	VWKNSSVQAF
SIV$_{gsn-99CM166}$	AAM90237	MMRKVGFLKM	LVVSLTLMAL	LIPGSRSKGN	WTTVFYGVPV
SIV$_{CPZ\ CAM5}$	CAB96414	MKAMERKKLN	WIICYMLMGL	ITPCLTGHEW	WATVYYGVPV

b

STRAIN	PEPTIDE
SIV$_{MAC239}$	MGCLGNQLL
	GNQLLIAIL
	NQLLIAILL
	QLLIAILLL
	SVCGIYCTL
SIV$_{MAC251}$	MGCLGNQLL
	GNQLLIAIL
	NQLLIAILL
	QLLIAILLL
SIV$_{AGM\ sab-1}$	KLLTVLLWL
	LWLSGCWSL
	SGCWSLVWL
SIV$_{AGM\ sab92018}$	KLLFLLVFL
	IGIVVIVKL
SIV$_{smSL92B}$	MACPGLHLL
	GLHLLIDIL
	IDILFLSVL
	LILWLVIGL
SIV$_{AGM.tan-1}$	GLGVGIVQL
SIV$_{gsn-99CM166}$	RKVGFLKML
	FLKMLVVSL
	KMLVVSLTL
	VVSLTLMAL
	VSLTLMALL
SIV$_{CPZ\ CAM5}$	MKAMERKKL
	LNWIICYML
	IICYMLMGL
	LMGLITPCL

c

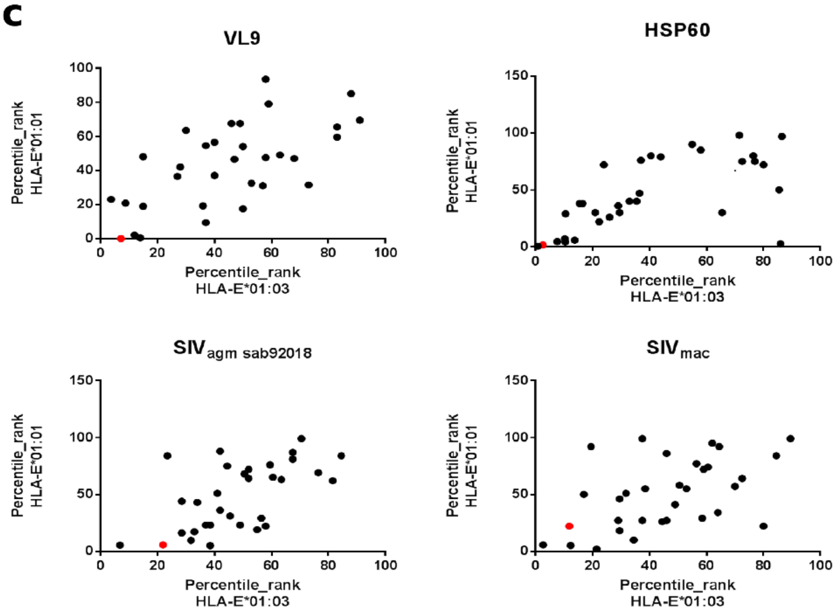

d

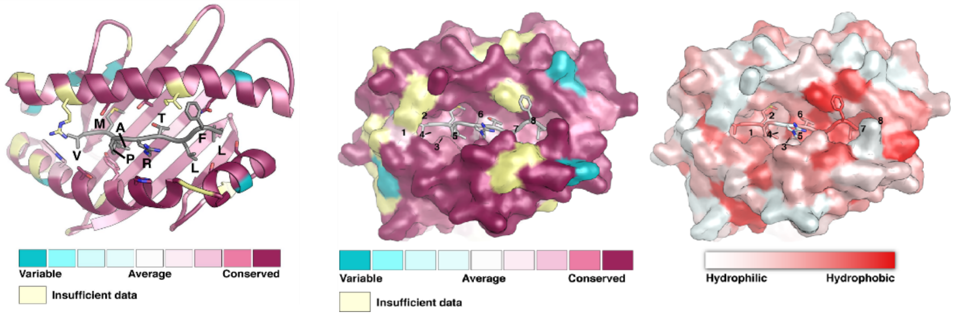

Supplementary Figure 10. Design of peptides derived from the ENV leader sequence with a potential for HLA-E binding. (a) Sequence alignments of the region corresponding to the signal peptides of the human HLA-class I B-73 alpha chain, the human HSP60 protein and the envelope proteins of 8 SIV viruses. The sequences of the human nonamer peptides (VL9, HSP60) known to efficiently bind to MHC-E are shown in red, as well as the positions of the Leucine amino acids. The sequences corresponding to the selected ENV peptides are framed. **(b)** Table showing nonamer sequences from signal peptides in ENV of the 8 analyzed SIV viruses showing similarities with the canonical MHC-E binding motif, such as strong hydrophobicity and a leucine in the last position. (**c)** HLA-E binding predictions using the IEDB analysis resource. This is a T cell epitope prediction tool, which takes into account an amino acid sequence and determines each subsequence's ability to bind to a specific MHC class I molecule[94,95]. Here the sequence corresponds to the leader sequence. The multiple possible subsequences of it are represented by the black dots in the graph. Each of the subsequence and its predicted capacity for binding to HLA-E is provided in the Source Data file. The location of each dot represents the predicted capacity of binding to HLA-E. For each peptide, percentile ranks for binding to HLA-E*0101 are indicated on the y-axis, and percentile ranks for binding to HLA-E*0103 are shown on the x-axis. A low percentile rank value indicates high affinity. The position of the known VL9 and HSP60 control peptides and of the selected SIV peptides are shown in red. The sequences for the selected SIVmac239 and SIVmac251 peptide are identical to each other. **(d)** Three-dimensional analysis of the binding pocket in the primate MHC-E molecule. Left and middle panel show the ribbon and surface representation of MHC-E with a peptide ligand (PDB code 3BZF) colored by degree of conservation based on the alignment of MHC-E amino acid sequences from *Homo sapiens*, *Pan troglodytes*, *Papio anubis*, *Chlorocebus sabaeus* (AGM), *Macaca mulatta* (rhMAC) and *Macaca fascicularis* (cynMAC). The MHC-E binding pocket was conserved between the species and the C-terminal binding part of the peptide highly hydrophobic for all species. The localization of a nonamer peptide with the canonical MHC-E binding motif is shown.

MAC					AGM			
Condition 1	Condition 2	Spearman r	P value		Condition 1	Condition 2	Spearman r	P value
NKG2a\clow CD16-	Cell-associated viral DNA for 10^6 cells	0,27605846	0,103156196		NKG2a\clow CD16-	Cell-associated viral DNA for 10^6 cells	0,18413949	0,282342693
NKG2a/c highCD16 -	Cell-associated viral DNA for 10^6 cells	-0,00825542	0,961887441		NKG2a/c highCD16 -	Cell-associated viral DNA for 10^6 cells	-0,4042192	0,01448192
NKG2a/c highCD16 +	Cell-associated viral DNA for 10^6 cells	0,33092575	0,048675578		NKG2a/c highCD16 +	Cell-associated viral DNA for 10^6 cells	-0,0834343	0,628539332
NKG2a\clow CD16+	Cell-associated viral DNA for 10^6 cells	-0,07186647	0,677029235		NKG2a\clow CD16+	Cell-associated viral DNA for 10^6 cells	0,45539944	0,005255768
NKG2a\clow CD16-	Cell-associated viral RNA for 10^6 cells	0,29205437	0,083917964		NKG2a\clow CD16-	Cell-associated viral RNA for 10^6 cells	0,14993068	0,389975072
NKG2a/c highCD16 -	Cell-associated viral RNA for 10^6 cells	-0,01186717	0,945234421		NKG2a/c highCD16 -	Cell-associated viral RNA for 10^6 cells	-0,3737374	0,026994524
NKG2a/c highCD16 +	Cell-associated viral RNA for 10^6 cells	0,37350099	0,024844214		NKG2a/c highCD16 +	Cell-associated viral RNA for 10^6 cells	-0,0033748	0,984649054
NKG2a\clow CD16+	Cell-associated viral RNA for 10^6 cells	-0,13934613	0,417632254		NKG2a\clow CD16+	Cell-associated viral RNA for 10^6 cells	0,43673004	0,008713923

Supplementary table 1: Correlation between NK cell subsets in LN and viral load in LN. The data on the NK cell subsets correspond to those shown in Figure 3b. The rho and p values of the correlations are shown.

List of the primers and probes used in the study				
**Primer**	**Sequence**	**Target**	**Gene**	**Purpose**
Forward	5'-ACCCAGTACAACAAATAGGTGGTAACT-3'	SIVmac251	GAG	quantification of plasma viral load of SIVmac251
Backward	5'-TCAATTTTACCCAGGCATTTAATGT-3'	SIVmac251	GAG	quantification of plasma viral load of SIVmac251
Probe	5'-6FAM-TGTCCACCTGCCATTAAGCCCGAG-3'-TAMRA	SIVmac251	GAG	quantification of plasma viral load of SIVmac251
Forward	5′-CTG GGT GTT CTC TGG TAA G-3′	SIVagm.Sab92018	LTR	quantification of plasma viral load of SIVagm.Sab92018
Backward	5′-CAA GAC TTT ATT GAG GCA AT-3′	SIVagm.Sab92019	LTR	quantification of plasma viral load of SIVagm.Sab92019
Probe	5'-6FAM-CGA ACA CCC AGG CTC AAG CTG G-3'-TAMRA	SIVagm.Sab92020	LTR	quantification of plasma viral load of SIVagm.Sab92020
Forward	5´-ACCCAGTACAACAAATAGGTGGTAACT-3´	SIVmac239	GAG	quantification of plasma viral load SIVmac239
Backward	5´-TCAATTTTACCCAGGCATTTAATGT-3´	SIVmac239	GAG	quantification of plasma viral load SIVmac239
Probe	5´-6FAM -TGTCCACCTGCCATTAAGCCCGAG-TAMRA-3'	SIVmac239	GAG	quantification of plasma viral load SIVmac239
VL9_sense	5'-caccctgctgctgGAACAATATGTTACAGTATTTTATG-3'	SIVagm.sab	ENV	Generation of SIVagm.sab mutant with e ENV LS sequence coding either for the nonamer peptide of VL9
VL9_Rev	5'-cgcggcgccatcacCAAAAAGACTAAAAGAAAAAGC-3'	SIVagm.sab	ENV	Generation of SIVagm.sab mutant with e ENV LS sequence coding either for the nonamer peptide of VL10
MAC239/251_sense	5'-cgccatcttgcttGAACAATATGTTACAGTATTTTATG-3'	SIVagm.sab	ENV	Generation of SIVagm.sab mutant with ENV LS sequence coding for the nonamer peptide of SIVmac239/251
MAC239/251_rev	5'-ataagcagctgattCAAAAAGACTAAAAGAAAAAGC-3'	SIVagm.sab	ENV	Generation of SIVagm.sab mutant with ENV LS sequence coding for the nonamer peptide of SIVmac239/252

Supplementary table 2: Primers used in the study.

Antibody	Clone	Suppliers	Cat number	Colors	Volume used to stain 10^6 cells (µl)	Control	Reference for Cross reactivity
CD3	SP34-2	BD biosciences	560772	V500	5	Isotype + FMO	Supplier and ref 1–5
CD45	D058-1283	BD biosciences	558411	Percp	5	Isotype + FMO	Supplier and ref 4,5
CD20	2H7	BD biosciences	560631	Alexa 700	4.5	Isotype + FMO	Supplier and ref 5–9
NKG2A/C	Z199	Beckman Coulter, Inc.	IM3291U	PE	8	Isotype + FMO	Supplier and ref 5,6,10
CD16	3G8	Beckman Coulter, Inc.	41116015	ECD	8	Isotype + FMO	Supplier and ref 5,6,9,10
CXCR5	710D82.1	NHP reagent	Na	PE	4	Isotype + FMO	Supplier and ref 11
CD8	BW135/80	Miltenyi	130-094-152	V450	2.5	Isotype + FMO	Supplier
CD4	L200	BD biosciences	560811	V450	2.5	Isotype + FMO	Supplier
CXCR3	1C6/CXCR3	BD biosciences	560831	PC7	5	Isotype + FMO	Supplier and ref 5,7,11,12
NKp80	4A4.D10	Miltenyi	130-094-843	FITC	6	Isotype + FMO	Supplier and ref 5,6,10
NKP30	AF29-4D12	Miltenyi	130-092-484	APC	6	Isotype + FMO	Supplier
CD279(PD-1)	EH12.2H7	Biolegend	329922	APC/cy7	5	Isotype + FMO	Supplier and ref 11
CD107a	H4A3	BD biosciences	555802	PC5	5	Isotype + FMO	Supplier
CD28	CD28.2	BD biosciences	562296	ECD	5	Isotype + FMO	Supplier
CD95	DX2	BD biosciences	561978	APC	5	Isotype + FMO	Supplier
CD69	FN50	BD biosciences	557756	APC/cy7	5	Isotype + FMO	Supplier and ref 3,12
HLA-DR	TU36	BD biosciences	551375	Alexa 700	6	Isotype + FMO	Supplier
PRF1	PF-344	mabtech	3465-71-100T	FITC	5	Isotype + FMO	Supplier
CCR7	3d12	Affymetrix eBioscience	11-1979-42	FITC	6	Isotype + FMO	Supplier
CD62L	DREG-56	biolegend	304808	PC5	6	Isotype + FMO	Supplier
CX3CR1	2a9-1	biolegends	341616	APC /cy7	6	Isotype + FMO	Supplier
CXCR5	MU5UBEE	Affymetrix eBioscience	15566616	FITC	5	Isotype + FMO	Supplier and ref 11
NKG2d	ON72	Beckman Coulter, Inc	A22329	APC	5	Isotype + FMO	Supplier
NKP46	BAB281	Beckman Coulter, Inc	A66902	PC5	6	Isotype + FMO	Supplier and ref 10
NKP44	2.29	Miltenyi	130-120-623	APC	6	Isotype + FMO	Supplier
Ki-67	MIB-1	Agilent	F726801-8	FITC	5	Isotype + FMO	Supplier
TNF-a	MAb11	BD biosciences	557996	Alexa 700	5	Isotype + FMO	Supplier
IFNg	45-15	Miltenyi	130-113-492	FITC	5	Isotype + FMO	Supplier and ref 6,13
CD14	M5E2	BD biosciences	557154	PE	5	Isotype + FMO	Supplier
GZMB	GB11	BD biosciences	560211	FITC	5	Isotype + FMO	Supplier and ref 6
TIM3	F38-2E2	biolegend	345012	APC	5	Isotype + FMO	Supplier
T-BET	O4-46	BD biosciences	561266	Alexa488	5	Isotype + FMO	Supplier

1. Nehete, P. N., Nehete, B. P., Chitta, S., Williams, L. E. & Abee, C. R. Phenotypic and Functional Characterization of Peripheral Blood Lymphocytes from Various Age- and Sex-Specific Groups of Owl Monkeys (Aotus nancymaae). Comp. Med.67, 67–78 (2017).
2. Foulds, K. E., Donaldson, M. & Roederer, M. OMIP-005: Quality and phenotype of antigen-responsive rhesus macaque T cells. Cytometry A81A, 360–361 (2012).
3. Sylwester, A. W., Hansen, S. G. & Picker, L. J. Quantification of T Cell Antigen-specific Memory Responses in Rhesus Macaques, Using Cytokine Flow Cytometry (CFC, also Known as ICS and ICCS): from Assay Set-up to Data Acquisition. Bio-Protoc.4, e1110–e1110 (2014).
4. Gaufin, T. et al. Experimental depletion of CD8+ cells in acutely SIVagm-Infected African Green Monkeys results in increased viral replication. Retrovirology7,42 (2010).
5. Huot, N. et al. Natural killer cells migrate into and control simian immunodeficiency virus replication in lymph node follicles in African green monkeys. Nat. Med.23, 1277–1286 (2017).
6. Weisgrau, K. L., Ries, M., Pomplun, N., Evans, D. T. & Rakasz, E. G. OMIP-035: Functional analysis of natural killer cell subsets in macaques. Cytometry A89, 799–802 (2016).
7. Palgen, J.-L. et al. NK cell immune responses differ after prime and boost vaccination. J. Leukoc. Biol.105, 1055–1073 (2019).
8. Amos, J. D. et al.Rapid Development of gp120-Focused Neutralizing B Cell Responses during Acute Simian Immunodeficiency Virus Infection of African GreenMonkeys. J. Virol.89, 9485–9498 (2015).
9. Sugimoto, C. et al. Differentiation Kinetics of Blood Monocytes and Dendritic Cells in Macaques: Insights to Understanding Human Myeloid Cell Development. J. Immunol. Baltim. Md 1950195, (2015).
10. Mavilio, D. et al. Identification of NKG2A and NKp80 as specific natural killer cell markers in rhesus and pigtailed monkeys. Blood106, 1718–1725 (2005).
11. Donaldson, M. M., Kao, S.-F. & Foulds, K. E. OMIP-052: An 18-Color Panel for Measuring Th1, Th2, Th17, and Tfh Responses in Rhesus Macaques. Cytometry A95, 261–263 (2019).
12. Jacquelin, B. et al. Innate immune responses and rapid control of inflammation in African green monkeys treated or not with interferon-alpha during primarySIVagm infection. PLoS Pathog.10, e1004241 (2014).
13. Hassan, W. M. et al. Multivariate profiling of African green monkey and rhesus macaque T lymphocytes. Sci. Rep.9, 4834 (2019).
14. Schafer, J. L., Li, H., Evans, T. I., Estes, J. D. & Reeves, R. K. Accumulation of Cytotoxic CD16+ NK Cells in Simian Immunodeficiency Virus-Infected Lymph Nodes Associated with In Situ Differentiation and Functional Anergy. J. Virol.89, 6887–6894 (2015).
15. Diop OM, Ploquin MJ, Mortara L, Faye A, Jacquelin B, Kunkel D, Lebon P, Butor C, Hosmalin A, Barré-Sinoussi F, Müller-Trutwin MC. Plasmacytoid dendritic cell dynamics and alpha interferon production during Simian immunodeficiency virus infection with a nonpathogenic outcome. J Virol. 2008 Jun;82(11):5145-52. Doi: 10.1128/JVI.02433-07. Epub 2008 Apr 2. PMID: 18385227; PMCID: PMC2395206

Supplementary table 3. Antibodies used for flow cytometry staining.
