# Supplementary material for: SIV-induced terminally differentiated adaptive NK cells in lymph nodes associated with enhanced MHC-E restricted activity
Source: Nat Commun. 2021 Feb 24;12:1282. doi: 10.1038/s41467-021-21402-1 (PMC7904927; doi:10.1038/s41467-021-21402-1)
Supplement: Supplementary file 3 — Reporting Summary [file 41467_2021_21402_MOESM3_ESM.pdf]

nature research

Corresponding author(s): Dr. Michaela Muller-Trutwin

Last updated by author(s): Jan 9, 2021

Reporting Summary

Nature Research wishes to improve the reproducibility of the work that we publish. This form provides structure for consistency and transparency in reporting. For further information on Nature Research policies, see our Editorial Policies and the Editorial Policy Checklist.

Statistics

For all statistical analyses, confirm that the following items are present in the figure legend, table legend, main text, or Methods section.

n/a	Confirmed	
☐	☒	The exact sample size (*n*) for each experimental group/condition, given as a discrete number and unit of measurement
☐	☒	A statement on whether measurements were taken from distinct samples or whether the same sample was measured repeatedly
☐	☒	The statistical test(s) used AND whether they are one- or two-sided<br>*Only common tests should be described solely by name; describe more complex techniques in the Methods section.*
☒	☐	A description of all covariates tested
☐	☒	A description of any assumptions or corrections, such as tests of normality and adjustment for multiple comparisons
☐	☒	A full description of the statistical parameters including central tendency (e.g. means) or other basic estimates (e.g. regression coefficient) AND variation (e.g. standard deviation) or associated estimates of uncertainty (e.g. confidence intervals)
☐	☒	For null hypothesis testing, the test statistic (e.g. *F*, *t*, *r*) with confidence intervals, effect sizes, degrees of freedom and *P* value noted<br>*Give P values as exact values whenever suitable.*
☒	☐	For Bayesian analysis, information on the choice of priors and Markov chain Monte Carlo settings
☒	☐	For hierarchical and complex designs, identification of the appropriate level for tests and full reporting of outcomes
☐	☒	Estimates of effect sizes (e.g. Cohen's *d*, Pearson's *r*), indicating how they were calculated

Our web collection on statistics for biologists contains articles on many of the points above.

Software and code

Policy information about availability of computer code

Data collection	Flow Cytometry data was collected using FACS DiVa software on an LSR II or LSR Fortessa (BD Biosciences). RNA-seq data was collected on an Illumina HiSeq 3000 system. Plasma viremia and cell-associated SIV-RNA and -DNA were collected using a Applied Biosystems 7500 Real-Time PCR.
Data analysis	Flow cytometry acquisitions were done on a LSRFortessa (BD Biosciences) using Diva software version (6.1.3) (build 2009 05 13 13 29) . The data were further analyzed using FlowJo 10.4.2 software (FlowJo, LLC, Ashland, OR, USA). Multiparametric analyses were performed using SPICE (version 5.1).<br>Bioinformatic analyses were performed using the RNA-seq pipeline from Sequana82. Reads were cleaned of adapter sequences, and low-quality sequences were removed using cutadapt version 1.1189. Only sequences ≥ 25 nucleotides (nt) in length were considered for further analysis. STAR version 2.5.0a, with default parameters, was used for alignment on the reference genome (Chlorocebus sabaeus, from Ensembl release 90). Genes were counted using featureCounts version 1.4.6-p390 from Subreads package (parameters: -t gene, -g ID and -s 1).<br>Data were analyzed using R version 3.4.3 and the Bioconductor package DESeq2 version 1.18.191.<br>Analyses and vizualization of GO terms associated with differentially expressed genes were performed using ClueGO version 2.5.4, a cytoscape plugin version 3.8.2.<br>Structures for rhesus, cynomolgus, African green monkey, baboon and chimpanzee proteins were modeled with the software MODELLER version 9.21, using the crystal structure of HLA-E*01:03 (PDB code 3BZF) as template. MHC-E amino acid sequence conservations were analyzed with the ConSurf server (Version 2.42 supporting JSmol (version 14.29.55_2019.10.30*) which is Java free viewer). Illustrations were rendered with PyMol version (2.4.1).<br>Pictures from confocal imaging were processed with ImageJ 1.51n with Java 1.8.0_66

For manuscripts utilizing custom algorithms or software that are central to the research but not yet described in published literature, software must be made available to editors and reviewers. We strongly encourage code deposition in a community repository (e.g. GitHub). See the Nature Research guidelines for submitting code & software for further information.

Data

Policy information about availability of data

All manuscripts must include a data availability statement. This statement should provide the following information, where applicable:

- Accession codes, unique identifiers, or web links for publicly available datasets
- A list of figures that have associated raw data
- A description of any restrictions on data availability

Deep sequencing results have been deposited in the Gene Expression Omnibus database; the accession number is GSE140600. https://www.ncbi.nlm.nih.gov/geo/query/acc.cgi?acc=GSE140600.
All the figures in the manuscript have been associated to the raw data files
Analyses and vizualization of GO terms associated with differentially expressed genes were performed using ClueGO version 2.5.4, a cytoscape plugin version 3.8.2. All the GOID and Ontology Source are provided in the raw data file.
The authors declare that all other data supporting the findings of this study are available from the authors upon request.

Field-specific reporting

Please select the one below that is the best fit for your research. If you are not sure, read the appropriate sections before making your selection.

- [x] Life sciences
- [ ] Behavioural & social sciences
- [ ] Ecological, evolutionary & environmental sciences

For a reference copy of the document with all sections, see nature.com/documents/nr-reporting-summary-flat.pdf

Life sciences study design

All studies must disclose on these points even when the disclosure is negative.

Sample size	The sample size varied between 3 and 9 monkeys per group (n = 6 in most experiments), chosen according to the tripartite harmonized International Council for Harmonization of Technical Requirements for Pharmaceuticals for Human Use (ICH) Guideline on Methodology (previously coded Q2B).
Data exclusions	No data were excluded from the analyses.
Replication	Some assays were performed as replicates: plasma viremia and cell-associated SIV-RNA/DNA quantifications and competitive analysis for binding of the peptides to MHC. The latter comprised three replicates (three independent experiments for each condition). We did not observe any major variation with all the replicates. All results were included in the manuscript. For the other assays, we only used biological replica (ie distinct animals). No other attempts were made at replication due to the limitation in volume of samples, and also duration of the in vivo study (2 years), substantial costs associated with animal acquisition, per diem charges, and surgical costs.
Randomization	Sample collection and analyses were performed in random order.
Blinding	The investigators were not blinded while the animal handlers were blinded to group allocation.

Reporting for specific materials, systems and methods

We require information from authors about some types of materials, experimental systems and methods used in many studies. Here, indicate whether each material, system or method listed is relevant to your study. If you are not sure if a list item applies to your research, read the appropriate section before selecting a response.

Materials & experimental systems

n/a	Involved in the study	
[ ]	[x]	Antibodies
[ ]	[x]	Eukaryotic cell lines
[x]	[ ]	Palaeontology and archaeology
[ ]	[x]	Animals and other organisms
[x]	[ ]	Human research participants
[x]	[ ]	Clinical data
[x]	[ ]	Dual use research of concern

Methods

n/a	Involved in the study	
[x]	[ ]	ChIP-seq
[ ]	[x]	Flow cytometry
[x]	[ ]	MRI-based neuroimaging

Antibodies

Antibodies used	Supplementary method table 1.

Validation	Supplementary method table 1.

Eukaryotic cell lines

Policy information about cell lines

Cell line source(s)	K562 cell line was provided by the ATCC ; The K-562 cell line was purchased from ATCC and transduced with a lentivirus expression vector for human HLA-E was purchases from Applied Biological Materials Inc.
Authentication	None of the cell lines were authenticated.
Mycoplasma contamination	we confirm that cell line was testes negative for mycoplasma
Commonly misidentified lines (See ICLAC register)	ATCC® Number: CCL-243™ ;no commonly misidentified cell lines were used in the study.

Animals and other organisms

Policy information about studies involving animals; ARRIVE guidelines recommended for reporting animal research

Laboratory animals	At the inclusion in the study the average weight of the monkeys was between 3 and 6 kg. All monkeys were young adults with an average age of 3–5 years at inclusion. Both males and females were used (60% females and 40% males for each species). Because H6 haplotypes are notably associated with viral control in cynomolgus macaques, macaques with H6 haplotype were excluded from this study.
Wild animals	Study did not involve wild animals.
Field-collected samples	Study did not involve field-collected samples
Ethics oversight	Animal experimental protocols were approved by the Ethical Committee of Animal Experimentation (CETEA-DSV, IDF, France) (Notification 12-098 and A17-044). The pVISCONTI study was approved and accredited under statement number A15-035 by the ethics committee “Comité d'Ethique en Expérimentation Animale du CEA”, registered and authorized under Number 2453-2015102713323361v2 by the French Ministry of Education and Research. The study at DPZ was approved by the Lower Saxony State Office for Consumer Protection and Food Safety and performed with the project licences 33.19-42502-04-12/0820 and 33.19-42502-04-17/2500. The DPZ has the permission to breed and house nonhuman primates under license number 392001/7 granted by the local veterinary office and conforming with § 11 of the German Animal Welfare act.

Note that full information on the approval of the study protocol must also be provided in the manuscript.

Flow Cytometry

Plots

Confirm that:

- [x] The axis labels state the marker and fluorochrome used (e.g. CD4-FITC).
- [x] The axis scales are clearly visible. Include numbers along axes only for bottom left plot of group (a 'group' is an analysis of identical markers).
- [x] All plots are contour plots with outliers or pseudocolor plots.
- [x] A numerical value for number of cells or percentage (with statistics) is provided.

Methodology

Sample preparation	Whole venous blood was collected in ethylenediaminetetraacetic acid (EDTA) tubes. Peripheral blood mononuclear cells (PBMCs) were isolated by Ficoll density-gradient centrifugation. Biopsies of peripheral lymph nodes (pLN) were performed by excision. Other tissues were collected at autopsy. After careful removal of adhering connective and fat tissues, LN and spleen cells were dissociated using the gentlemacS™ Dissociator technology (Miltenyi Biotec, Germany). The cell suspension was subsequently filtered through 100- and 40-µm cell strainers, and cells were washed with cold phosphate-buffered saline (PBS). Cells were either immediately stained for flow cytometry or cryopreserved in 90% foetal bovine serum (FBS) and 10% dimethyl sulfphoxide (DMSO) and stored in liquid nitrogen before use. Intracellular staining was performed using BD Cytofix/ Cytoperm™.
Instrument	Flow cytometry acquisitions were done on a LSRFortessa (BD Biosciences).
Software	The data were analysed using Diva (software version 6.1.3, build 2009 05 13 13 29). The data were further analyzed using FlowJo (10.4.2 software, LLC, Ashland, OR, USA). Multiparametric analyses were performed using SPICE (version 5.1). t-SNE was performed with the cytobank (Cytobank, Inc.), using 2,000 iterations and a perplexity of 60.
Cell population abundance	Online methods, paragraph: Polychromatic flow cytometry ; Cell sorting of NK cell sub-populations.

Gating strategy | Supplementary Figure 1.

☒ Tick this box to confirm that a figure exemplifying the gating strategy is provided in the Supplementary Information.
